# Exome sequencing points to pathogenic *ATM* variants in gastric cancer

**DOI:** 10.1038/s41431-025-01994-8

**Published:** 2025-12-26

**Authors:** Laura L. Koebbe, Timo Hess, Stephan L. Haas, Ines Gockel, Guillaume Piessen, Anna Latiano, Carina Pereira, Ewa Malecka-Wojciesko, Anna Mokrowiecka, Stefania Boccia, Marek Majewski, Hakan Alakus, Ángel Lanas, Roberta Pastorino, Thorsten Oliver Goetze, Peter Elbe, Nicole Kreuser, Orazio Palmieri, Francesca Tavano, Christiane Josephine Bruns, Olivier Glehen, Xavier B. D’Journo, Caroline Gronnier, Jean M. Fabre, Laurent Sulpice, Luis Bujanda, Leticia Moreira, Stefanie Heilmann-Heimbach, Maximilian Billmann, Markus M. Noethen, Renato Cannizzaro, Michele Ghidini, Lutz Hamann, Nuria Aragones, Mário Dinis-Ribeiro, Rui Medeiros, Salah-Eddin Al-Batran, Mārcis Leja, Juozas Kupcinskas, María A. García-González, Carlo Maj, Marino Venerito, Johannes Schumacher

**Affiliations:** 1 Center for Human Genetics, Philipps-Universität Marburg, Marburg, Germany; 2 Department of Upper GI Diseases, Karolinska University Hospital and Unit of Gastroenterology and Rheumatology, Karolinska Institutet, Stockholm, Sweden; 3 Department of Visceral, Transplant, Thoracic and Vascular Surgery, University Hospital Leipzig, Leipzig, Germany; 4 University Lille, CNRS, Inserm, CHU Lille, UMR9020-U1277 - CANTHER - Cancer Heterogeneity, Plasticity and Resistance to Therapies, Lille, France; 5 Gastroenterology Unit, Fondazione IRCCS Casa Sollievo della Sofferenza, San Giovanni Rotondo, Italy; 6 Precancerous Lesions and Early Cancer Management Group (CI-IPOP), Portuguese Oncology Institute of Porto (IPO Porto) / Porto Comprehensive Cancer Center – Raquel Seruca (Porto.CCC) & CI-IPOP@RISE (Health Research Network), Porto, Portugal; 7 Department of Digestive Tract Diseases, Medical University of Lodz, Lodz, Poland; 8 Department of Woman and Child Health and Public Health - Public Health Area, Fondazione Policlinico Universitario A. Gemelli IRCCS, Roma, Italia; 9 Section of Hygiene, University Department of Life Sciences and Public Health, Università Cattolica del Sacro Cuore, Roma, Italia; 10 2nd Department of General Surgery, Medical University of Lublin, Lublin, Poland; 11 Department of General, Visceral, Cancer and Transplant Surgery, University of Cologne, Cologne, Germany; 12 Centro de Investigación Biomédica en Red en Enfermedades Digestivas y Hepáticas (CIBERehd), Madrid, Spain; 13 Department of Gastroenterology, Hospital Clínico Universitario Lozano Blesa, Zaragoza, Spain; 14 Instituto Aragonés de Ciencias de la Salud (IACS), Zaragoza, Spain; 15 Krankenhaus Nordwest, University Cancer Center, Frankfurt, Germany; 16 Institut für Klinische Krebsforschung IKF GmbH am Krankenhaus Nordwest, Frankfurt, Germany; 17 Department of Digestive Surgery, Hôpital Hospitalier Lyon Sud, Hospices Civils de Lyon, Lyon, France; 18 Department of Thoracic Surgery, Aix-Marseille University, Assistance Publique-Hôpitaux de Marseille, Marseille, France; 19 Department of Digestive Surgery, Haut-Lévêque Hospital, Bordeaux, France; 20 Department of Digestive Surgery, Centre Hospitalier Universitaire de Montpellier, Montpellier, France; 21 Department of Digestive, Hepatobiliary Surgery and Liver Transplantation, Pontchaillou Hospital, CHU Rennes, Rennes, France; 22 Department of Gastroenterology, Hospital Donostia/Instituto Biodonostia, Universidad del País Vasco (UPV/EHU), San Sebastián, Spain; 23 Department of Gastroenterology, Hospital Clinic of Barcelona, Institut d’Investigacions Biomediques August Pi i Sunyer (IDIBAPS), Barcelona, Spain; 24 Institute of Human Genetics, University of Bonn, Bonn, Germany; 25 NGS Core Facility, Medical Faculty, University of Bonn, Bonn, Germany; 26 Unit of Oncological Gastroenterology, Centro di Riferimento Oncologico di Aviano (CRO) IRCCS, Aviano, Italia; 27 Department of Medical, Surgical and Health Sciences, University of Trieste, Trieste, Italy; 28 Medical Oncology Unit, ASST of Cremona, Cremona, Italy; 29 Medical Oncology Unit, Fondazione IRCCS Ca’ Granda Ospedale Maggiore Policlinico, Milan, Italy; 30 Institute of Microbiology, Infectious Diseases and Immunology, Charité – Universitätsmedizin Berlin, corporate member of Freie Universität Berlin and Humboldt-Universität zu Berlin, Berlin, Germany; 31 Consortium for Biomedical Research in Epidemiology & Public Health (CIBER en Epidemiología y Salud Pública e CIBERESP), Madrid, Spain; 32 Epidemiology Section, Public Health Division, Department of Health of Madrid, Madrid, Spain; 33 Gastroenterology Department, Portuguese Institute of Oncology of Porto, Porto, Portugal; 34 Molecular Oncology and Viral Pathology Group (CI-IPOP), Portuguese Oncology Institute of Porto (IPO Porto) / Porto Comprehensive Cancer Center – Raquel Seruca (Porto.CCC) & CI-IPOP@RISE (Health Research Network), Porto, Portugal; 35 Research Department of the Portuguese League Against Cancer-North (LPCC-NRNorte), Porto, Portugal; 36 Riga East University Hospital, Riga, Latvia; 37 Institute of Clinical and Preventive Medicine, University of Latvia, Riga East University Hospital, Riga, Latvia; 38 Digestive Diseases Centre GASTRO, Riga, Latvia; 39 Gastroenterology Department and Institute for Digestive Research, Lithuanian University of Health Sciences, Kaunas, Lithuania; 40 Instituto de Investigación Sanitaria Aragón (IIS Aragón), Zaragoza, Spain; 41 Department of Gastroenterology, Hepatology and Infectious Diseases, Otto-von-Guericke University Hospital, Magdeburg, Germany

**Keywords:** Genetics research, Stomach, Cancer genetics, Gastrointestinal cancer, Risk factors

## Abstract

Gastric cancer (GC) is a leading cause of cancer-related deaths worldwide. While most cases result from the cumulative risk of common genetic variants, a smaller proportion shows a monogenic etiology. We used germline exome sequencing data to assess the gene-based burden of loss-of-function pathogenic variants (LoF-PVs) in 471 early-onset GC cases as well as 666 GC cases from the UK Biobank (UKB), aiming to identify monogenic GC forms. In both datasets, LoF-PVs in *CDH1* and *ATM* were enriched among GC cases. Beyond GC, *ATM* LoF-PVs were also enriched in UKB participants with pancreatic, oesophageal, breast, prostate, and lung cancer, though the effect size was notably high in GC. As an established disease gene for pancreatic, breast, ovarian, and prostate cancer, *ATM* should also be considered for genetic testing when monogenic GC is suspected. This is especially important for families in which other tumours associated with *ATM* PVs occur alongside GC.

## Introduction

The etiology of gastric adenocarcinoma, here called gastric cancer (GC), is heterogeneous. Most cases have a multifactorial etiology, with common genetic variants conferring disease risk cumulatively. In contrast, a small proportion of GC cases have a monogenic etiology, driven by highly penetrant pathogenic variants (PVs) in single genes. Accordingly, monogenic GC forms show a familial aggregation. Furthermore, they are characterised by an early age at onset (AAO) and a higher prevalence of other cancers in affected families. Referring to the American Society of Clinical Oncology (ASCO) [1] and the guideline from the National Comprehensive Cancer Network (NCCN) for GC [2], 11 genes are currently recommended for testing if a monogenic tumour syndrome involving GC is suspected. These are hereditary diffuse gastric cancer with PVs in *CDH1* and *CTNNA1* [3], Lynch syndrome with PVs in *MLH1*, *MSH2*, *MSH6*, *PMS2*, and *EPCAM*, and polyposis syndromes with PVs in *BMPR1A*, *SMAD4*, *STK11*, and *APC* [1, 2]. Additionally, GC occurs as part of several other hereditary cancer predisposition syndromes, including Li-Fraumeni syndrome caused by PVs in *TP53*, hereditary breast and ovarian cancer with PVs in *BRCA1* and *BRCA2*, *MUTYH*-associated polyposis syndrome, and *PTEN* hamartoma tumour syndrome [4]. The present study aimed to identify novel monogenic GC genes.

## Materials and methods

### Sample description

In a multicenter study, we recruited 4885 European individuals with histopathologically confirmed GC [5]. No first-degree relative was reported with GC or other cancers suggestive of monogenic tumour syndromes. Nevertheless, the presence of such syndromes cannot be excluded entirely due to de novo mutations in patients, incomplete penetrance, variable expressivity, or unreported positive family history. Of all patients, 501 (199 females, 302 males) with an AAO below 55 years (mean ± SD: 44 ± 6.3 years) underwent exome sequencing (ES) to identify novel monogenic GC forms. Tumour histopathology based on the Lauren classification was diffuse in 206, intestinal in 120, and mixed in 47 patients, while histopathology data was unavailable for the remaining 128 cases. *Helicobacter pylori* status was only available for a subset of the cohort. Sample characteristics are summarised in Supplementary Table 1.

### ES and variant calling

ES was conducted on early-onset GC samples using the Twist Human Comprehensive Exome Kit (Twist Bioscience, San Francisco, CA, USA). Exome-enriched libraries were sequenced as 2 × 150 bp paired-end reads on a NovaSeq 6000 system (Illumina, San Diego, CA, USA), achieving approximately 100 x coverage. Germline variants were detected with the nf-core/sarek pipeline [6], where reads were aligned to the Genome Reference Consortium human reference 37 assembly (GRCh37/hg19) using BWA-MEM [7]. Joint calling of single nucleotide variants and small insertions/deletions across all samples was performed using GATK HaplotypeCaller [8].

### Sample and variant quality control

Samples were filtered for call rate, heterozygosity, sex discordance, relatedness, and European ancestry, leaving 471 GC cases for analysis. Indels were left-aligned and multiallelic sites split into biallelic records. Variants were filtered by call rate, allele balance, and Hardy-Weinberg equilibrium. Those located in low-complexity regions or short tandem repeats were excluded. Details on the quality control (QC) can be found in the Supplementary Information.

### Gene-based burden testing

Gene-based burden testing with CoCoRV [9] was performed on post-QC ES data from 471 GC cases, using 51,377 cancer-free European individuals from the Genome Aggregation Database (gnomAD) v2.1.1 [10] as controls. Rare loss-of-function (LoF) PVs with a minor allele frequency (MAF) < 1% in both cases and controls were aggregated per gene, with allele counts combined across cases and controls. GC candidate genes were defined by Bonferroni-corrected exome-wide significance (*P* < 2.5 ×10^-6^, corresponding to 0.05/20,000 protein-coding genes), a minimum of 5 LoF-PV heterozygotes in the GC cohort, and not being commonly mutated. Accordingly, the 100 genes exhibiting the highest frequency of rare non-synonymous or splice-site variants in public exomes, as determined by Shyr et al. [11], were not considered. Genes meeting the filtering criteria and their corresponding *P* values are provided in Supplementary Table 2. All variants in the identified GC candidate genes were classified according to American College of Medical Genetics and Genomics (ACMG) guidelines [12] and their annotation was confirmed with VariantValidator [13]. Further details on QC and variant filtering can be found in the Supplementary Information.

For replication, we conducted gene-based burden testing using ES data from the population-based UK Biobank (UKB; study ID 135122). Sample and variant QC for this dataset was performed by the UKB. We additionally excluded samples with non-European ancestry, sex discordance, and third-degree relatives (kinship coefficient > 0.0442), yielding in a final set of 372,587 samples. This included 666 GC cases identified by ICD10 diagnosis code C16 (malignant neoplasm of stomach) in medical records. GC enrichment testing targeted rare (MAF < 1%) LoF-PVs in three candidate genes identified in the discovery step (*CDH1*, *ATM*, *FANCA*), along with 15 previously reported genes associated with tumour syndromes involving GC. Statistical analyses used two-sided Fisher’s exact tests with a Bonferroni-corrected significance threshold of *P* < 2.78 ×10^-3^ to account for all 18 analysed genes. Nominal *P* values below 0.05 were considered as suggestive evidence.

### Cancer phenotyping of *ATM* LoF-PV heterozygotes among UKB participants

To further characterise the cancer spectrum associated with *ATM* LoF-PVs, we utilised UKB ES data. Enrichment testing was conducted for 21 solid tumours among individuals harbouring *ATM* LoF-PVs using ICD10 codes to classify cancer types. Statistical analyses were performed using two-sided Fisher’s exact tests with Bonferroni correction for multiple comparisons (Bonferroni-corrected *P* < 2.38 × 10^-3^ to account for 21 tested cancers).

## Results

Our discovery gene-based burden analysis identified three potential monogenic GC genes (*CDH1*, *ATM*, *FANCA*) with an enrichment of LoF-PVs among cases (Table 1). *CDH1* showed the most significant enrichment of LoF-PVs (6 LoF-PV heterozygotes, *P* = 3.78 ×10^-12^), followed by *ATM* (7 LoF-PV heterozygotes, *P* = 6.41 ×10^-9^), and *FANCA* (5 LoF-PV heterozygotes, *P* = 7.41 ×10^-9^). The tumour histopathology was diffuse in all *CDH1* LoF-PV heterozygotes, with an average AAO of 36.5 years (Table 1). Most *ATM* LoF-PV heterozygotes had intestinal GC (*N* = 5) and the average AAO was 46.6 years (Table 1). Among *FANCA* LoF-PV heterozygotes, tumour types were diffuse (N = 3), intestinal (N = 1) or unknown (N = 1) with a mean AAO of 43.4 years (Table 1). All LoF-PVs in *CDH1* and *ATM* were classified as pathogenic or likely pathogenic based on ACMG criteria [12], whereas one LoF-PV in *FANCA* was of uncertain significance. Among the established GC genes[1, 2], we identified one LoF-PV in *CTNNA1* in addition to *CDH1*, both associated with hereditary diffuse gastric cancer, but no PVs in genes associated with other tumour syndromes involving GC (data not shown).

**Table 1:** Identification of gastric cancer (GC)-associated genes and pathogenic variant (PV) annotation.

	Cases		Controls						
Gene	LoF +	LoF -	LoF +	LoF -	*P*				
***CDH1***	6	465	1	51,376	3.78 ×10^-12^				
	**Sample**	**Nucleotide change**	**Amino acid change**	**Effect**	**ACMG classification**	**ACMG evidence**	**Lauren classification**	**AAO**	**mean AAO**
	FR-GC-169	c.187 C > T	p.Arg63Ter	stopgain	P	PVS1, PM2_Supporting, PS4_Supporting, PM5_Supporting	diffuse	31	36.5
	LV-GC-656	c.220 C > T	p.Arg74Ter	stopgain	P	PVS1, PM2_Supporting, PS4_Supporting, PM5_Supporting	diffuse	32	
	GE-GC-290	c.244_248del	p.Val82TyrfsTer10	frameshift deletion	LP	PVS1, PM2_Supporting	diffuse	23	
	FR-GC-027	c.385 C > T	p.Gln129Ter	stopgain	LP	PVS1, PM2_Supporting	diffuse	42	
	SP-GC-043	c.1914G>A	p.Trp638Ter	stopgain	LP	PVS1, PM2_Supporting	diffuse	51	
	GE-GC-325	c.2386del	p.Arg796GlyfsTer20	frameshift deletion	LP	PVS1, PM2_Supporting	diffuse	40	
	**Cases**		**Controls**						
**Gene**	**LoF +**	**LoF -**	**LoF +**	**LoF -**	***P***				
***ATM***	7	464	22	51,355	6.41 ×10^-9^				
	**Sample**	**Nucleotide change**	**Amino acid change**	**Effect**	**ACMG classification**	**ACMG evidence**	**Lauren classification**	**AAO**	**mean AAO**
	GE-GC-118	c.802 C > T	p.Gln268Ter	stopgain	P	PVS1, PM2_Supporting, PM5_Supporting	diffuse	47	46.6
	GE-GC-371	c.1564_1565del	p.Glu522IlefsTer43	frameshift deletion	LP	PVS1, PM5_Supporting	intestinal	46	
	GE-GC-298	c.1573 A > T	p.Lys525Ter	stopgain	P	PVS1, PM2_Supporting, PM5_Supporting	intestinal	51	
	LV-GC-639	c.2554 C > T	p.Gln852Ter	stopgain	P	PVS1, PM2_Supporting, PM5_Supporting	intestinal	45	
	GE-GC-456	c.3802del	p.Val1268Ter	stopgain	LP	PVS1, PM5_Supporting	mixed	41	
	LT-GC-127	c.5932 G > T	p.Glu1978Ter	stopgain	LP	PVS1, PM5_Supporting	intestinal	45	
	GE-GC-059	c.7327 C > T	p.Arg2443Ter	stopgain	P	PVS1, PM2_Supporting, PM5_Supporting	intestinal	51	
	**Cases**		**Controls**						
**Gene**	**LoF +**	**LoF -**	**LoF +**	**LoF -**	***P***				
***FANCA***	5	466	4	51,373	7.41 ×10^-9^				
	**Sample**	**Nucleotide change**	**Amino acid change**	**Effect**	**ACMG classification**	**ACMG evidence**	**Lauren classification**	**AAO**	**mean AAO**
	IT-GC-024	c.275 C > A	p.Ser92Ter	stopgain	LP	PVS1, PM2_Supporting	intestinal	40	43.4
	SP-GC-160	c.295 C > T	p.Gln99Ter	stopgain	LP	PVS1, PM2_ Supporting	diffuse	48	
	LV-GC-660	c.3634_3640del	p.Ser1212ArgfsTer33	frameshift deletion	LP	PVS1, PM2_ Supporting	diffuse	44	
	SW-GC-016	c.4069_4082del	p.Ala1357LeufsTer63	frameshift deletion	LP	PVS1, PM2_ Supporting	unknown	47	
	FR-GC-137	c.4309 C > T	p.Gln1437Ter	stopgain	VUS*	PVS1_Moderate, PM2_ Supporting	diffuse	38	

* Note: Although this variant showed limited pathogenicity evidence based on ACMG classification, it was still included in the gene-based burden analysis, as ACMG criteria were not applied to filter variants.

Burden analysis was conducted using 471 GC cases and 51,377 cancer-free European controls from the Genome Aggregation Database (gnomAD). Two-sided Fisher’s exact tests revealed significant GC associations for loss-of-function- (LoF-) PVs in *CDH1*, *ATM*, and *FANCA*. Nominal *P* values are shown. Statistical significance was assessed using a Bonferroni correction to account for multiple testing. Considering 20,000 protein-coding genes, the corrected significance threshold is *P* < 2.5 × 10^-6^. LoF-PVs were annotated with nucleotide and amino acid changes relative to RefSeq transcripts. The predicted functional effects and classifications of PVs based on guidelines from the American College of Medical Genetics and Genomics (ACMG): P (pathogenic), LP (likely pathogenic), and VUS (variant of uncertain significance) [12]. Clinical data of GC cases included Lauren classification and age at onset (AAO). AAO was available for all cases, while Lauren classification was missing for one. Odds ratios have not been computed as gnomAD controls are hypernormal cancer-free individuals and, thus, not representative of the population-based relative risk. Sample prefixes indicate the country of origin: GE Germany, FR France, IT Italy, LT Lithuania, LV Latvia, SP Spain, SW Sweden.

*AAO* age at onset, *ACMG* American College of Medical Genetics and Genomics, *LoF* loss-of-function.

The replication analysis utilised ES data of 372,587 participants from the population-based UKB, including 666 GC cases. In the UKB cohort, we observed a significant enrichment of GC cases among LoF-PV heterozygotes in *ATM* (odds ratio [OR], 3.94; 95% confidence interval [CI], 1.69-7.84; *P* = 1.26 ×10^-3^) and a nominally significant enrichment in *CDH1* (OR, 11.2; 95% CI, 1.34–41.72; *P* = 0.015), while no GC enrichment was found among *FANCA* LoF-PV heterozygotes (Table 2). Among previously reported GC-associated genes, only LoF-PVs in *MSH2* and *APC* showed nominal associations with GC (Table 2).

**Table 2:** Exome sequencing (ES) data analysis of 372,587 UK Biobank participants including 666 gastric cancer (GC) cases.

	GC +		GC -			
Gene	LoF +	LoF -	LoF +	LoF -	OR (95% CI)	*P*
***ATM***	8	658	1143	370,778	3.94 (1.69-7.84)	1.26 ×10^-3^
***MSH2***	3	663	182	371,739	9.24 (1.88-27.56)	4.64 ×10^-3^
***CDH1***	2	664	100	371,821	11.2 (1.34-41.72)	0.015
***APC***	2	664	133	371,788	8.42 (1.01-31.14)	0.025
***CTNNA1***	1	665	61	371,860	9.17 (0.23-53.17)	0.105
***EPCAM***	3	663	643	371,278	2.61 (0.54-7.7)	0.111
***MSH6***	5	661	1857	370,064	1.51 (0.49-3.54)	0.396
***PMS2***	3	663	1130	370,791	1.48 (0.3-4.37)	0.461
***FANCA***	6	660	2594	369,327	1.29 (0.47-2.84)	0.479
***MUTYH***	1	665	379	371,542	1.47 (0.04-8.29)	0.493
***BRCA2***	17	649	8141	363,780	1.17 (0.68-1.89)	0.506

Enrichment testing for GC was performed in heterozygotes for loss-of-function (LoF)-pathogenic variants (PVs) for 3 candidate genes identified in our ES study (*CDH1*, *ATM*, *FANCA*) as well as 15 previously reported genes associated with tumour syndromes involving GC. GC cases were defined by ICD10 code C16 (malignant neoplasm of stomach) in medical records. “GC +” denotes persons with GC, “GC -“ those without. “LoF +” indicates LoF-PV heterozygotes, “LoF -” individuals without LoF-PV. Two-sided Fisher’s exact tests revealed significant GC enrichment among LoF-PV heterozygotes for *ATM*. Nominal *P* values are shown. Statistical significance was assessed using a Bonferroni correction to account for multiple testing. Considering 16 known GC-predisposing genes as well as *ATM* and *FANCA*, the corrected significance threshold is *P* < 2.78 × 10^-3^. No GC cases with LoF-PVs in *BMPR1A*, *BRCA1*, *MLH1*, *PTEN*, *SMAD4, STK11*, or *TP53* were observed. Therefore, these seven genes are not listed. The average age at onset was not provided, as the UKB is a population-based prospective study focusing on diseases of middle and older age, and therefore lacks representation of early-onset cancer cases.

*CI* confidence interval, *GC* gastric cancer, *LoF* loss-of-function, *OR* odds ratio.

To assess the tumour spectrum related to *ATM* LoF-PVs, we tested the enrichment of 21 solid cancer types among *ATM* LoF-PV heterozygotes in the UKB. Six cancer types were enriched among *ATM* LoF-PV heterozygotes (Table 3), with LoF-PVs in *ATM* contributing strongest to pancreatic cancer based on the effect size (OR, 6.96; 95% CI, 3.94–11.42; *P* = 4.23 ×10^-9^), followed by esophageal cancer (OR, 4.23; 95% CI, 2.01–7.64; *P* = 9.4 ×10^-5^) and GC (OR, 3.94; 95% CI, 1.69–7.84; *P* = 1.26 ×10^-3^) (Table 3). Notably, all of these cancers belong to the upper gastrointestinal tract. In contrast, the effect sizes for lung, breast, and prostate cancer, that also showed significant enrichments of *ATM* LoF-PVs, were comparably smaller (OR < 2.7) (Table 3).

**Table 3:** Enrichment testing for 21 solid tumours conducted in *ATM* loss-of-function- (LoF-) pathogenic variant (PV) heterozygotes from UK Biobank.

		ICD10 +		ICD10 -			
ICD10	Cancer	*ATM* LoF +	*ATM* LoF -	*ATM* LoF +	*ATM* LoF -	OR (95% CI)	*P*
C25	Pancreas	16	751	1135	370,685	6.96 (3.94-11.42)	4.23 ×10^-9^
C50	Breast	67	10,354	1084	361,082	2.16 (1.66-2.76)	4.48 ×10^-8^
C61	Prostate	44	6895	1107	364,541	2.1 (1.52-2.84)	1.44 ×10^-5^
C15	Oesophagus	11	845	1140	370,591	4.23 (2.1-7.64)	9.40 ×10^-5^
C34	Bronchus/lung	21	2590	1130	368,846	2.65 (1.63-4.07)	9.48 ×10^-5^
C16	Stomach	8	658	1143	370,778	3.94 (1.69-7.84)	1.26 ×10^-3^
C22	Liver/liver ducts	5	433	1146	371,003	3.74 (1.21-8.82)	0.012
C56	Ovary	7	1015	1,144	370,421	2.23 (0.89-4.63)	0.042
C18	Colon	17	3388	1134	368,048	1.63 (0.94-2.62)	0.059
C67	Bladder	13	2382	1138	369,054	1.77 (0.94-3.04)	0.06
C55	Uterus	2	138	1149	371,298	4.68 (0.56-17.28)	0.07
C43	Melanoma	11	2261	1140	369,175	1.58 (0.78-2.84)	0.128
C20	Rectum	8	1606	1143	369,830	1.61 (0.69-3.19)	0.172
C23	Gallbladder	1	78	1150	371,358	4.14 (0.1-23.8)	0.217
C73	Thyroid gland	2	385	1149	371,051	1.68 (0.2-6.12)	0.336
C54	Corpus uteri	2	1344	1149	370,092	0.48 (0.06-1.74)	0.454
C53	Cervix uteri	1	237	1150	371,199	1.36 (0.03-7.68)	0.521
C62	Testis	1	243	1150	371,193	1.33 (0.03-7.49)	0.53
C64	Kidney	4	1163	1147	370,273	1.11 (0.3-2.86)	0.787
C71	Brain	1	556	1150	370,880	0.58 (0.01-3.25)	1
C32	Larynx	0	252	1151	371,184	0 (0-4.76)	1

ICD10 codes were used for cancer classification. “ICD10 +” indicates the presence of an ICD10 cancer diagnosis, “ICD10 -” its absence. “LoF +” denotes *ATM* LoF-PV heterozygotes, “LoF -” individuals without LoF-PV. Statistical analyses used two-sided Fisher’s exact tests, suggesting a significant association of *ATM* LoF-PVs with pancreas, breast, prostate, oesophagus, lung, and gastric cancers. Nominal *P* values are shown. Statistical significance was assessed using a Bonferroni correction to account for multiple testing. Considering 21 tested cancers, the corrected significance threshold is *P* < 2.38 × 10^-3^.

*CI* confidence interval, *LoF* loss-of-function, *OR* odds ratio.

## Discussion

GC is genetically heterogeneous [5]. While most cases are sporadic and have a polygenic etiology, there are hereditary cancer syndromes that predispose to monogenic GC forms. By investigating the burden of LoF-PVs in 471 early-onset cases, we aimed to uncover novel monogenic GC genes. This study represents the largest ES-based investigation of early-onset GC in the European population to date.

In our discovery sample, we identified monogenic forms of GC in approximately 3% of early AAO cases (13/471), referring to *CDH1* and *ATM*. *CDH1* represents the established GC gene leading to hereditary diffuse gastric cancer [1], consistent with the diffuse tumour histopathology observed in all *CDH1* LoF-PV heterozygotes. In contrast, *ATM* is not an established monogenic GC gene [1], although PVs in *ATM* have been previously reported in GC patients [14–22]. In our sample, *ATM* LoF-PV heterozygotes predominantly presented with intestinal GC. Future studies need to determine whether this histopathology is specifically linked to *ATM* PVs, as prior studies did not report such an association, likely due to few GC patients with *ATM* PVs or limited histopathological data [14–22]. While *FANCA* emerged as monogenic GC candidate gene in our discovery sample and in another GC ES study [23], this association was not replicated in the UKB. This lack of replication may be explained by the small proportion of early-onset GC cases in the UKB. Thus, larger early-onset cohorts are required to determine whether *FANCA* represents another predisposition gene for monogenic GC.

According to ASCO guidelines, *ATM* is an established disease gene for pancreatic, breast, ovarian, and prostate cancer, but not for GC [1]. Assessing the tumour spectrum related to *ATM* LoF-PVs in the UKB, we found an enrichment across six cancer types, with the largest effect sizes in pancreatic and esophageal cancers, followed by GC. Thus, our study − together with findings from previous studies [14–22] − imply that *ATM* should be considered in clinical testing when a monogenic form of GC is suspected. This is particularly relevant in cases of early-onset GC and/or in families where tumours belonging to the spectrum of *ATM* PVs, especially other cancers of the upper gastrointestinal tract, are observed alongside GC.

## Supplementary information

Supplementary Information

Supplementary Table 1

Supplementary Table 2

## Data Availability

The ES data is publicly available at Sequence Read Archive (SRA), BioProject ID PRJNA1168527 (https://www.ncbi.nlm.nih.gov/bioproject/PRJNA1168527). The gnomAD v.2.1.1 summary level ES data can be downloaded from https://gnomad.broadinstitute.org/downloads#v2. The UKB ES data is accessible via the UK Biobank Research Analysis Platform (UKB-RAP) via https://ukbiobank.dnanexus.com/.

## Code availability

Variant detection was performed using the nf-core/sarek pipeline (https://nf-co.re/sarek/3.2.3/). Quality control filtering of VCF files was performed using PLINK (v1.90b7), gatk (v4.4.0.0), bedtools (v2.31.0) bcftools (v1.19), and vcftools (0.1.16). Gene-based burden analysis was performed using CoCoRV v1.1 (https://bitbucket.org/Wenan/cocorv/src/master/). Data manipulation and analysis of the UKB ES data was performed using R 4.1.2 with the packages ukbtools (v0.11.3), dplyr (v1.1.3) and stringr (v1.5.1). The code for each of the R packages can be found in their associated vignettes.

## References

[1] Tung N, Ricker C, Messersmith H, Balmana J, Domchek S, Stoffel EM, et al. Selection of Germline Genetic Testing Panels in Patients With Cancer: ASCO Guideline. J Clin Oncol. 2024;42:2599–615. 38759122 10.1200/JCO.24.00662

[2] National Comprehensive Cancer Network. Gastric Cancer (Version 2.2024). 2024. http://www.nccn.org/professionals/physician_gls/pdf/gastric.pdf.

[3] Blair VR, McLeod M, Carneiro F, Coit DG, D’Addario JL, van Dieren JM, et al. Hereditary diffuse gastric cancer: updated clinical practice guidelines. Lancet Oncol. 2020;21:e386–e97. 32758476 10.1016/S1470-2045(20)30219-9 PMC7116190

[4] Carneiro F. Familial and hereditary gastric cancer, an overview. Best Pract Res Clin Gastroenterol. 2022;58-59:101800. 35988963 10.1016/j.bpg.2022.101800

[5] Hess T, Maj C, Gehlen J, Borisov O, Haas SL, Gockel I, et al. Dissecting the genetic heterogeneity of gastric cancer. EBioMedicine. 2023;92:104616. 37209533 10.1016/j.ebiom.2023.104616 PMC10212786

[6] Garcia M, Juhos S, Larsson M, Olason PI, Martin M, Eisfeldt J, et al. Sarek: A portable workflow for whole-genome sequencing analysis of germline and somatic variants. F1000Res. 2020;9:63. 32269765 10.12688/f1000research.16665.1 PMC7111497

[7] Li H Aligning sequence reads, clone sequences and assembly contigs with BWA-MEM. arXiv. 2013.

[8] Poplin R, Ruano-Rubio V, DePristo MA, Fennell TJ, Carneiro MO, Van der Auwera GA, et al. Scaling accurate genetic variant discovery to tens of thousands of samples. bioRxiv. 2017;201178.

[9] Chen W, Wang S, Tithi SS, Ellison DW, Schaid DJ, Wu G. A rare variant analysis framework using public genotype summary counts to prioritize disease-predisposition genes. Nat Commun. 2022;13:2592. 35545612 10.1038/s41467-022-30248-0 PMC9095601

[10] Karczewski KJ, Francioli LC, Tiao G, Cummings BB, Alfoldi J, Wang Q, et al. The mutational constraint spectrum quantified from variation in 141,456 humans. Nature. 2020;581:434–43. 32461654 10.1038/s41586-020-2308-7 PMC7334197

[11] Shyr C, Tarailo-Graovac M, Gottlieb M, Lee JJ, van Karnebeek C, Wasserman WW. FLAGS, frequently mutated genes in public exomes. BMC Med Genomics. 2014;7:64. 25466818 10.1186/s12920-014-0064-y PMC4267152

[12] Richards S, Aziz N, Bale S, Bick D, Das S, Gastier-Foster J, et al. Standards and guidelines for the interpretation of sequence variants: a joint consensus recommendation of the American College of Medical Genetics and Genomics and the Association for Molecular Pathology. Genet Med. 2015;17:405–24. 25741868 10.1038/gim.2015.30 PMC4544753

[13] Freeman PJ, Hart RK, Gretton LJ, Brookes AJ, Dalgleish R. VariantValidator: Accurate validation, mapping, and formatting of sequence variation descriptions. Hum Mutat. 2018;39:61–68. 28967166 10.1002/humu.23348 PMC5765404

[14] Carvalho, Oliveira J, Senz P, Sao Jose J, Hansford S C, Teles SP, et al. Redefinition of familial intestinal gastric cancer: clinical and genetic perspectives. J Med Genet. 2021;58:1–11. 32066632 10.1136/jmedgenet-2019-106346

[15] Guadagnolo D, Mastromoro G, Marchionni E, Germani A, Libi F, Sadeghi S, et al. Heterozygous Pathogenic Nonsense Variant in the ATM Gene in a Family with Unusually High Gastric Cancer Susceptibility. Biomedicines. 2023;11:2062. 10.3390/biomedicines11072062 PMC10377208 37509701

[16] Hall MJ, Bernhisel R, Hughes E, Larson K, Rosenthal ET, Singh NA, et al. Germline Pathogenic Variants in the Ataxia Telangiectasia Mutated (ATM) Gene are Associated with High and Moderate Risks for Multiple Cancers. Cancer Prev Res. 2021;14:433–40. 10.1158/1940-6207.CAPR-20-0448 PMC8026745 33509806

[17] Helgason H, Rafnar T, Olafsdottir HS, Jonasson JG, Sigurdsson A, Stacey SN, et al. Loss-of-function variants in ATM confer risk of gastric cancer. Nat Genet. 2015;47:906–10. 26098866 10.1038/ng.3342

[18] Huang DS, Tao HQ, He XJ, Long M, Yu S, Xia YJ, et al. Prevalence of deleterious ATM germline mutations in gastric cancer patients. Oncotarget. 2015;6:40953–8. 26506520 10.18632/oncotarget.5944 PMC4747381

[19] Slavin T, Neuhausen SL, Rybak C, Solomon I, Nehoray B, Blazer K, et al. Genetic Gastric Cancer Susceptibility in the International Clinical Cancer Genomics Community Research Network. Cancer Genet. 2017;216-217:111–9. 29025585 10.1016/j.cancergen.2017.08.001 PMC5659836

[20] Tedaldi G, Pirini F, Tebaldi M, Zampiga V, Cangini I, Danesi R, et al. Multigene Panel Testing Increases the Number of Loci Associated with Gastric Cancer Predisposition. Cancers. 2019;11:1340. 10.3390/cancers11091340 PMC6769562 31514334

[21] Guerra J, Estrada-Florez AP, Lott PC, Pinto C, Pinheiro M, Chiu KA, et al. The role of multi-organ cancer predisposition genes in the risk of inherited and histologically diverse gastric cancer. EBioMedicine. 2025;116:105759. 40446793 10.1016/j.ebiom.2025.105759 PMC12166715

[22] Usui Y, Taniyama Y, Endo M, Koyanagi YN, Kasugai Y, Oze I, et al. Helicobacter pylori, Homologous-Recombination Genes, and Gastric Cancer. N Engl J Med. 2023;388:1181–90. 36988593 10.1056/NEJMoa2211807

[23] Nurgalieva A, Galliamova L, Ekomasova N, Yankina M, Sakaeva D, Valiev R, et al. Whole Exome Sequencing Study Suggests an Impact of FANCA, CDH1 and VEGFA Genes on Diffuse Gastric Cancer Development. Genes. 2023;14:280. 10.3390/genes14020280 PMC9956399 36833207

