# Supplementary material for: Exome sequencing points to pathogenic ATM variants in gastric cancer
Source: Eur J Hum Genet. 2025 Dec 26;34(9):1311–6. doi: 10.1038/s41431-025-01994-8 (PMC13550501; doi:10.1038/s41431-025-01994-8)
Supplement: Supplementary file 1 [file 41431_2025_1994_MOESM1_ESM.docx]

**Exome sequencing points to pathogenic *ATM* variants in gastric cancer**

Laura L Koebbe,^1,*^ Timo Hess,^1,*^ Stephan L Haas,^2^ Ines Gockel,^3^ Guillaume Piessen,^4^ Anna Latiano,^5^ Carina Pereira,^6^ Ewa Malecka-Wojciesko,^7^ Anna Mokrowiecka,^7^ Stefania Boccia,^8,9^ Marek Majewski,^10^ Hakan Alakus,^11^ Ángel Lanas,^12,13,14^ Roberta Pastorino,^8^ Thorsten Oliver Goetze,^15,16^ Peter Elbe,^2^
Nicole Kreuser,^3^ Orazio Palmieri,^5^ Francesca Tavano,^5^ Christiane Josephine Bruns,^11^ Olivier Glehen,^17^
Xavier B D’Journo,^18^ Caroline Gronnier,^19^ Jean M Fabre,^20^ Laurent Sulpice,^21^ Luis Bujanda,^12,22^
Leticia Moreira,^12,23^ Stefanie Heilmann-Heimbach,^24,25^ Maximilian Billmann,^24^ Markus M Noethen,^24^ Renato Cannizzaro,^26,27^ Michele Ghidini,^28,29^ Lutz Hamann,^30^ Nuria Aragones,^31,32^ Mário Dinis-Ribeiro,^6,33^ Rui Medeiros,^34,35^
Salah-Eddin Al-Batran,^15,16^ Mārcis Leja,^36,37,38^ Juozas Kupcinskas,^39^ María A García-González,^12,14,40^
Carlo Maj,^1,§^ Marino Venerito,^41,§^ Johannes Schumacher^1,§^

1 Center for Human Genetics, University of Marburg, Marburg, Germany
2 Department of Upper GI Diseases, Karolinska University Hospital and Unit of Gastroenterology and Rheumatology, Karolinska Institutet, Stockholm, Sweden
3 Department of Visceral, Transplant, Thoracic and Vascular Surgery, University Hospital Leipzig, Leipzig, Germany
4 University Lille, CNRS, Inserm, CHU Lille, UMR9020-U1277 - CANTHER - Cancer Heterogeneity, Plasticity and Resistance to Therapies, Lille, France
5 Gastroenterology Unit, Fondazione IRCCS Casa Sollievo della Sofferenza, San Giovanni Rotondo, Italy
6 Precancerous Lesions and Early Cancer Management Group (CI-IPOP), Portuguese Oncology Institute of Porto (IPO Porto) / Porto Comprehensive Cancer Center – Raquel Seruca (Porto.CCC) & CI-IPOP@RISE (Health Research Network), Porto, Portugal
7 Department of Digestive Tract Diseases, Medical University of Lodz, Lodz, Poland
8 Department of Woman and Child Health and Public Health - Public Health Area, Fondazione Policlinico Universitario A. Gemelli IRCCS, Roma, Italia
9 Section of Hygiene, University Department of Life Sciences and Public Health, Università Cattolica del Sacro Cuore, Roma, Italia
10 2nd Department of General Surgery, Medical University of Lublin, Lublin, Poland
11 Department of General, Visceral, Cancer and Transplant Surgery, University of Cologne, Cologne, Germany
12 Centro de Investigación Biomédica en Red en Enfermedades Digestivas y Hepáticas (CIBERehd), Madrid, Spain
13 Department of Gastroenterology, Hospital Clínico Universitario Lozano Blesa, Zaragoza, Spain
14 Instituto Aragonés de Ciencias de la Salud (IACS), Zaragoza, Spain
15 Krankenhaus Nordwest, University Cancer Center, Frankfurt, Germany
16 Institut für Klinische Krebsforschung IKF GmbH am Krankenhaus Nordwest, Frankfurt, Germany
17 Department of Digestive Surgery, Hôpital Hospitalier Lyon Sud, Hospices Civils de Lyon, Lyon, France
18 Department of Thoracic Surgery, Aix-Marseille University, Assistance Publique-Hôpitaux de Marseille, Marseille, France
19 Department of Digestive Surgery, Haut-Lévêque Hospital, Bordeaux, France
20 Department of Digestive Surgery, Centre Hospitalier Universitaire de Montpellier, Montpellier, France
21 Department of Digestive, Hepatobiliary Surgery and Liver Transplantation, Pontchaillou Hospital, CHU Rennes, Rennes, France
22 Department of Gastroenterology, Hospital Donostia/Instituto Biodonostia, Universidad del País Vasco (UPV/EHU), San Sebastián, Spain
23 Department of Gastroenterology, Hospital Clinic of Barcelona, Institut d'Investigacions Biomediques August Pi i Sunyer (IDIBAPS), Barcelona, Spain
24 Institute of Human Genetics, University of Bonn, Bonn, Germany
25 NGS Core Facility, Medical Faculty, University of Bonn, Bonn, Germany
26 Unit of Oncological Gastroenterology, Centro di Riferimento Oncologico di Aviano (CRO) IRCCS, Aviano, Italia
27 Department of Medical, Surgical and Health Sciences, University of Trieste, Trieste, Italy
28 Medical Oncology Unit, ASST of Cremona, Cremona, Italy
29 Medical Oncology Unit, Fondazione IRCCS Ca' Granda Ospedale Maggiore Policlinico, Milan, Italy
30 Institute of Microbiology, Infectious Diseases and Immunology, Charité – Universitätsmedizin Berlin, corporate member of Freie Universität Berlin and Humboldt-Universität zu Berlin, Berlin, Germany
31 Consortium for Biomedical Research in Epidemiology & Public Health (CIBER en Epidemiología y Salud Pública e CIBERESP), Spain
32 Epidemiology Section, Public Health Division, Department of Health of Madrid, Madrid, Spain
33 Gastroenterology Department, Portuguese Institute of Oncology of Porto, Porto, Portugal
34 Molecular Oncology and Viral Pathology Group (CI-IPOP), Portuguese Oncology Institute of Porto (IPO Porto) / Porto Comprehensive Cancer Center – Raquel Seruca (Porto.CCC) & CI-IPOP@RISE (Health Research Network), Porto, Portugal
35 Research Department of the Portuguese League Against Cancer-North (LPCC-NRNorte), Porto, Portugal
36 Riga East University Hospital, Riga, Latvia
37 Institute of Clinical and Preventive Medicine, University of Latvia, Riga East University Hospital, Riga, Latvia
38 Digestive Diseases Centre GASTRO, Riga, Latvia
39 Gastroenterology Department and Institute for Digestive Research, Lithuanian University of Health Sciences, Kaunas, Lithuania
40 Instituto de Investigación Sanitaria Aragón (IIS Aragón), Zaragoza, Spain
41 Department of Gastroenterology, Hepatology and Infectious Diseases, Otto-von-Guericke University Hospital, Magdeburg, Germany

* These authors contributed equally to this work.

^§^ These authors jointly supervised this work.

**SUPPLEMENTARY METHODS**

**Sample quality control (QC)**

From the exome sequencing data, variants with a sequencing read depth ≥ 20x in 85% of samples were retained. Samples were excluded if their missing call rate or autosomal heterozygosity rate deviated more than 6 standard deviations above the mean. Samples with sex discordance, indicated by an F-statistic > 0.2 for females or < 0.8 for males, were also removed. To eliminate related individuals up to the third degree, kinship coefficients were calculated using KING^1^ software. For individuals with a kinship coefficient > 0.442, the sample with the lower call rate was excluded. Ancestry outliers from the European population were identified using principal component analysis with the 1,000 Genomes Phase 3 data^2^. Samples whose Euclidean distance from the European reference population's means of the first two PCs exceeded 1.25 times the maximum Euclidean distance of the European reference samples were removed. Further exclusions applied to samples with an excessive number of unknown variants with respect to dbSNPv.150 (median + 6 median absolute deviations (MAD) for single nucleotide variants, median + 12 MAD for indels), aberrant heterozygous/homozygous ratios (median ± 6 MAD), or low transition/transversion ratios (median - 6 MAD). After QC, 471 individuals with gastric cancer remained for analysis.

**Variant QC**

In variant QC, indels were left-aligned, multiallelic sites were split into biallelic records and analyses were restricted to variants in autosomal protein-coding genes. Variants located in low-complexity regions or short tandem repeats were excluded. Additional criteria removed variants with an average allele balance < 0.25 or > 0.75 for heterozygous calls or < 0.9 for homozygous calls, with a call rate < 0.9 and those where the genotype frequencies significantly deviated from Hardy Weinberg equilibrium (P < 1 x 10^-6^).

**Gene-based burden testing**

The CoCoRV^3^ pipeline was used to prioritize GC risk genes enriched for rare loss-of-function (LoF) PVs via gene-based burden testing. To ensure compatibility between case data and summary-control datasets, consistent variant filtering was applied so that only high-quality variants were included. For both cases and controls, analyses were restricted to regions where at least 90% of samples had sequencing depth ≥10. To minimize false positives, a curated set of genomic regions and variants prone to calling errors, including low-complexity regions and segmental duplications, was excluded. Variants failing QC in either cases or controls based on GATK VQSR filter status were also removed. Rare variants in high linkage disequilibrium were identified and excluded. During normalization, indels were standardized, and multi-allelic variants were split into separate biallelic variants. Genotypes were set to missing if they did not meet the minimum thresholds for depth of coverage (DP) ≥10 and genotype quality (GQ) ≥20. Additionally, heterozygous calls were required to have an allele fraction between 0.2 and 0.8. All variants were functionally annotated using ANNOVAR^4^. Those annotated as LoF, including frameshift deletion and insertions, stopgain, and splicing variants, with a combined MAF in cases and controls below 1% were retained for the burden analysis. Gene-based burden testing was conducted under a dominant model, comparing cases and controls harboring at least one qualifying variant in a gene to those without, using Fisher’s exact test. A flowchart visualizing the QC steps and variant filtering in the gene-based burden analysis is shown in Supplementary Figure 1.

**Supplementary Figure 1: Workflow from sequencing data to high-confidence variants used for analysis.** Description of filtering and QC steps in gene-based burden testing with CoCoRV.

DP, depth of coverage; ES, exome sequencing; GATK, genome analysis toolkit; GQ, genotype quality; LoF, loss-of-function; MAF, minor allele frequency; VAF, variant allele frequency; VQSR, variant quality score recalibration

**REFERENCES**

1.	Manichaikul A, Mychaleckyj JC, Rich SS, Daly K, Sale M, Chen WM. Robust relationship inference in genome-wide association studies. Bioinformatics. 2010;26:2867-2873.

2.	Auton A, Brooks LD, Durbin RM, Garrison EP, Kang HM, Korbel JO*, et al.* A global reference for human genetic variation. Nature. 2015;526:68-74.

3.	Chen W, Wang S, Tithi SS, Ellison DW, Schaid DJ, Wu G. A rare variant analysis framework using public genotype summary counts to prioritize disease-predisposition genes. Nat Commun. 2022;13:2592.

4.	Wang K, Li M, Hakonarson H. ANNOVAR: functional annotation of genetic variants from high-throughput sequencing data. Nucleic Acids Res. 2010;38:e164.
